# Robotic surgery in colorectal emergencies: a systematic review of current evidence

**DOI:** 10.1186/s13017-026-00685-z

**Published:** 2026-05-08

**Authors:** Serena Curia, Christophe Taoum, Guglielmo Niccolò Piozzi, Diana Ronconi Di Giuseppe, Abhijeet Beniwal, Sentilnathan Subramaniam, Fausto Catena, Micaela Piccoli, Jim S. Khan

**Affiliations:** 1 Department of Colorectal Surgery, Portsmouth Hospitals University NHS Trust, Queen Alexandra Hospital, Southwick Hill Road, Cosham, Portsmouth, PO6 3LY UK; 2 Department of General, Emergency Surgery and New Technologies, Baggiovara General Hospital, Azienda Ospedaliero Universitaria Di Modena, Modena, Italy; 3 Department of Surgical Oncology, Montpellier Cancer Institute (Institut du Cancer de Montpellier, ICM), Montpellier, France; 4 Faculty of Science and Health, University of Portsmouth, Portsmouth, UK; 5 Department of Surgery, Alma Mater Studiorum, University of Bologna, Bologna, Italy; 6 Colorectal Surgery, St. Mark’s Hospital at Central Middlesex Hospital, London, UK; 7 Colorectal Surgery Unit, Department of General Surgery, Hospital Queen Elizabeth, Kota Kinabalu, Malaysia; 8 Department of General, Emergency, and Trauma Surgery, “M. Bufalini” Hospital, Cesena, Italy

**Keywords:** Robotic, Emergency surgery, Colorectal surgery, Colorectal emergencies, Colorectal cancer, Diverticular disease

## Abstract

**Background:**

Although laparoscopy continues to be the predominant minimally invasive approach in most emergency settings, the advantages of robotics, well established in elective surgery, are currently being explored in selected scenarios and specialized centres.

**Methods:**

A systematic review was conducted using PubMed, Cochrane Library and Scopus databases until January 2025. Primary outcome was safety and feasibility of robotics in emergency colorectal surgery. Secondary endpoints included perioperative and postoperative outcomes.

**Results:**

Fifteen articles were included with a total of 46 robotic emergency colorectal surgical procedures. Most were performed in a tertiary centre with a da Vinci system. Most common procedures were robotic right hemicolectomy for colon cancer and sigmoid colectomy for acute diverticulitis. Mean operating time for robotic right hemicolectomy was 134 min for benign cases and 241 ± 7 min for malignant cases; robotic sigmoid colectomy showed a mean operating time of 171 ± 3 min. No intraoperative complications were recorded. One case required conversion. Intracorporeal anastomosis was performed in most cases (n = 13). Mean length of stay was 5 days. No Clavien-Dindo grade ≥ 3 complications, reoperation or readmission were reported. Five complete mesocolic excisions (CMEs) were performed. Pathology outcomes were available for four CMEs: showing R0 resection with a mean lymph node harvest of 54 ± 13. In four CMEs, the involved team included an on-call robotic colorectal surgeon and an experience theatre team including experienced anaesthetist in robotic procedures.

**Conclusions:**

Robotics in emergency settings is feasible and safe but requires additional training and dedicated teams for optimal outcomes.

**Supplementary Information:**

The online version contains supplementary material available at 10.1186/s13017-026-00685-z.

## Introduction

Robotic approach is widely used worldwide across various surgical specialties, primarily for elective procedures. Globally, around 17 million robotic procedures have been performed using the Intuitive Surgical (Sunnyvale, CA, USA) robotic systems alone [1]. Laparoscopy has been widely adopted for emergency cases due to better short-term outcomes (reduced postoperative pain, shorter length of stay (LOS), lower risk of surgical site infection and overall complications, and decreased blood loss) than traditional open surgery. There are limited reports regarding the use of robotic surgery in emergencies. Lately, the World Society of Emergency Surgery (WSES) position paper has suggested that the emergency setting should not be considered as a contraindication for robotic surgery provided that an adequately trained team is available [2].

Pedraza et al. [3] described the first robotic emergency surgery in 2012. Since then, robotics has been adopted in emergency settings at referral centres, but its use remains limited and may present certain challenges, particularly for teams that are not adequately trained in using the robot in this setting. This is also influenced by general concerns on safety of the robotic approach, despite the availability of protocols for emergency undocking [4, 5]. Other factor that contributes to the limited use of robotics in emergency surgery is the availability of a dedicated robotic platform and a trained robotic team throughout the day. Most centres have one or few robotic systems that are mostly utilised for elective procedures during office hours, leading to emergency procedures to be performed laparoscopically. On the contrary, with limited trained robotic team personnel, performing emergency robotic surgery after hours on a regular basis could be unsafe, suboptimal, and cause potential mental and physical burnout resulting in a decline in overall performance with harm on the patient. Expert anaesthetist support is advocated in maintaining the patients in a stable hemodynamic state to allow a robotic approach as a viable surgical option in emergency setting, due to the longer procedure than laparotomy. Further concerns involve the associated higher costs and longer operative times of robotics, compared to laparoscopy, as well as the learning curve [6].

However, the rapid rise in robotic adoption, reinforced by the national healthcare system 10-year plan to ensure that 90% of minimally invasive abdominal surgeries will be performed robotically by 2035, accelerates the widespread integration of this approach, pushing the boundaries of surgical innovation in the emergency setting.

This study aims to review the status and applications of robotics in colorectal emergency surgery for benign and malignant diseases.

## Materials and methods

### Study design and literature research

The protocol was registered on PROSPERO (CRD42024620565). The systematic review was conducted according to the 2020 PRISMA statement (Preferred Reporting Items for Systematic reviews and Meta-Analyses) guidelines [7]. The PRISMA checklist is reported as Supplementary File 1. Two authors (S.C. and C.T.) performed the search strategy independently on PubMed, Cochrane Library, and Scopus databases, including articles from January 2000 until January 2025. The electronic search was limited only to human studies in English language and conducted according to the following keywords: “robotic emergencies, emergency surgery, robotic colorectal surgery, colorectal emergencies, colorectal cancer, diverticular disease”. Rayyan software was used (https://www.rayyan.ai) to collate the data.

### PICOS

(P)opulation: all patients undergoing robotic emergency colorectal surgery**;** (I)ntervention: robotic colorectal surgical procedures in emergency setting**;** (C)omparison: not applicable**;** (O)utcome: safety and feasibility of robotic emergency colorectal surgery**;** (S)tudies: all types of studies, no restrictions were applied.

### Eligibility criteria

Inclusion criteria were: (1) Studies on robotic emergency colorectal surgery (2) Robotic approach; (3) Adult population (≥ 18 years); (4) Emergency setting (clinical scenario requiring surgery within 24 h in stable patients with a low risk of deterioration) or urgent setting (condition requiring surgery within 72 h in stable patients, but not suitable for discharge) [8]. Exclusion criteria were: (1) Other surgical approaches; (2) Appendectomies and hernias; (3) Articles published in languages other than English.

### Aim

Primary aim was to evaluate the safety and feasibility of robotic approach in emergency colorectal surgery. Secondary aims were to assess robotic approach, surgical technique, perioperative and postoperative outcomes after robotic emergency colorectal surgery. Postoperative complications were graded according to the Clavien-Dindo classification [9].

### Methodological quality appraisal

Results of the critical assessment for each study are reported in Table 1 according to the Joanna Briggs Institute (JBI) Risk of Bias Assessment [10]. Two studies were of low quality (4/10). The remaining studies were of moderate quality, scoring 5 to 7 points. The main methodological limitations included inappropriate statistical analysis (86%), lack of a statement on consecutive patient inclusion and complete participant inclusion (100%), unclear reporting of patients' clinical information (43%), and incomplete reporting of outcomes or follow-up results (33%).

**Table 1:** Quality appraisal (risk of bias) for case series, and case control (*)

Publication, year	Clear criteria for inclusion	Condition measured in a standard, reliable way	Valid methods used for identification of the condition	Consecutive inclusion	Complete inclusion	Clear reporting of the demographics	Clear reporting of clinical information	Outcomes or follow up results	Clear reporting of the presenting site/clinic demographic information	Statistical analysis appropriate	Total
Pedraza et al., 2012	Y	Y	Y	U	U	Y	Y	Y	Y	U	7/10
Smith et al., 2023	Y	Y	Y	U	U	Y	Y	U	Y	U	6/10
Alhammadi et al.,2024	Y	Y	Y	U	U	Y	Y	Y	Y	U	7/10
Alhomaid et al., 2024	Y	Y	Y	U	U	Y	Y	Y	Y	U	7/10
Felli et al.,2014	Y	Y	Y	U	U	Y	Y	Y	Y	U	7/10
Jambhekar et al.,2018	Y	Y	Y	U	U	Y	Y	Y	Y	U	7/10
Monsellato et al., 2019	Y	Y	Y	U	U	Y	Y	Y	Y	U	7/10
Kudsi et al.,2019	Y	Y	Y	U	U	Y	Y	Y	Y	U	7/10
Kudsi et al.,2020	Y	Y	Y	U	U	Y	U	U	Y	U	5/10
Kudsi et al.,2020	Y	Y	Y	U	U	Y	U	U	Y	U	5/10
De’Angelis et al.,2024	Y	Y	Y	U	U	Y	U	U	Y	U	5/10
Sneddon et al.,2023	Y	Y	Y	U	U	Y	U	U	Y	U	5/10
Maertens et al.,2022	Y	U	U	U	U	Y	N	Y	Y	Y	5/10
Ceccarelli et al.,2024	Y	U	U	U	U	Y	N	Y	N	Y	4/10
Petropoulou et al., 2025	Y	U	U	U	U	Y	N	Y	Y	U	4/10

Y yes; N; no; U uncertain not applicable

### Statistical analysis

Categorical data were expressed as absolute values and/or pooled percentages. Continuous data were expressed as absolute mean with standard deviation for normally distributed data or median values with interquartile ranges for non-normally distributed data. Statistical analyses were conducted using IBM SPSS Statistics for Windows, version 28 (IBM Corp., Armonk, NY, USA).

## Results

### Studies characteristics and patients demographics

**Fig. 1:**
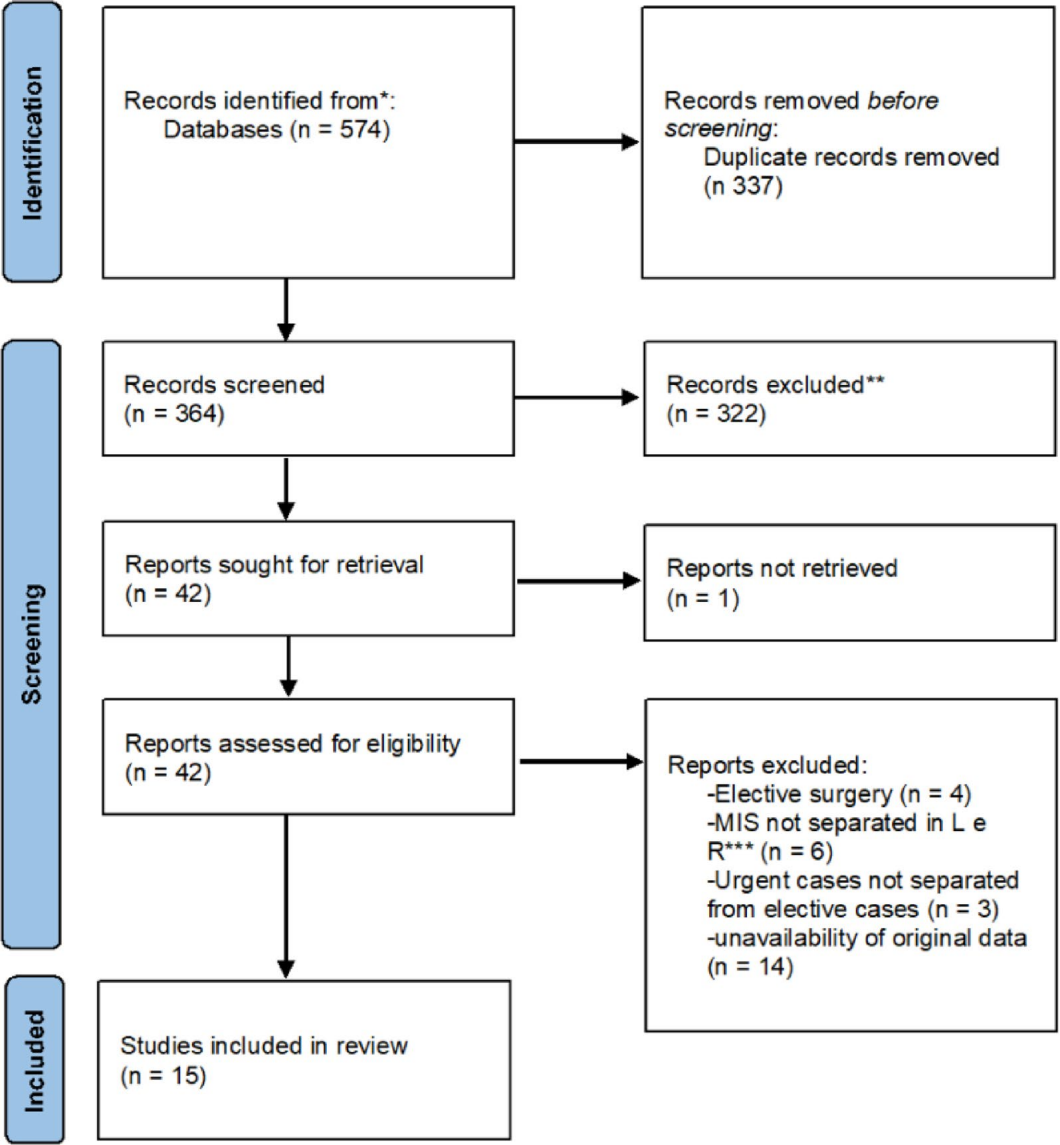
Prisma flow diagram. *Consider, if feasible to do so, reporting the number of records identified from each database or register searched (rather than the total number across all databases/registers). **If automation tools were used, indicate how many records were excluded by a human and how many were excluded by automation tools. *** Laparoscopic surgery (L); Robotic surgery (R)

The initial literature search yielded 574 publications related to emergent and urgent robotic colorectal surgery. After removing duplicates (n = 337), 364 unique articles underwent title and abstract screening, resulting in 42 studies selected for full-text review. Of these, 27 were excluded due to not meeting inclusion criteria (n = 13) or inaccessibility of original patient-specific data (n = 14). Corresponding authors of these inaccessible studies were contacted via email, however after three unsuccessful contact attempts, their exclusion was confirmed. Reference lists of included articles were screened to identify additional eligible studies. Any discrepancies during study selection were resolved through discussion, with final decisions made by consensus among reviewers. Finally, 15 studies were included: three case series [8, 11, 12], five video-vignettes [13–17], and seven case-reports [3, 18–23]. Of these, ten studies were conducted in Europe, four in USA, and one in the United Arab Emirates. The study inclusion process is reported in Fig. 1.

A total of 46 surgical procedures were analysed. Most patients were male (n = 28, 61%), with a mean age of 66 ± 14 years. Mean body mass index (BMI) was 26 ± 3 kg/m^2^. Regarding American Society of Anesthesiologists (ASA) classification, seven patients (15%) were ASA ≥ 3, 22 (48%) were ASA < 3, whilst the ASA status was not specified in 17 cases (37%). Patients’ demographics are summarized in Table 2.

**Table 2:** Demographics

Publication, year	Study design	Country	Age	Gender	BMI Kg/m2	ASA score	Previous abdominal surgery
Pedraza et al., 2012	Case report	USA	84	Female	22.1	II	Open total abdominal hysterectomy
Smith et al., 2023	Case report	USA	74	Male	NA	NA	NA
Alhammadi et al., 2024	Case report	UAE	55	Male	30.85	NA	Unremarkable
Alhomaid et al., 2024	Case report	USA	80	Female	NA	NA	Appendectomy
Felli et al., 2014	Case report	France	86	Female	22	NA	NA
Jambhekar et al., 2018	Case report	USA	74	Male	NA	NA	Unremarkable
Monsellato et al., 2019	Case report	Italy	74	Male	NA	NA	No
Kudsi et al., 2019	Video vignette	Lithuania	69	Female	25	II	NA
Kudsi et al., 2020	Video vignette	Lithuania	69	Female	19	III	NA
Kudsi et al., 2020	Video vignette	UK	81	Female	38	III	NA
De’Angelis et al., 2024	Video vignette	Italy	60	Male	NA	NA	NA
Sneddon et al., 2023	Video vignette	UK	50	Female	NA	NA	NA
Maertens et al., 2022	Case series	UK	83	Female	22	II	NA
Maertens et al., 2022	Case series	UK	76	Female	19	III	NA
Maertens et al., 2022	Case series	UK	36	Female	41	II	NA
Maertens et al., 2022	Case series	UK	74	Male	24	II	NA
Maertens et al., 2022	Case series	UK	59	Male	24	II	NA
Maertens et al., 2022	Case series	UK	51	Male	22	II	NA
Maertens et al., 2022	Case series	UK	74	Male	27	II	NA
Maertens et al., 2022	Case series	UK	75	Male	26	III	NA
Maertens et al., 2022	Case series	UK	71	Male	33	III	NA
Maertens et al., 2022	Case series	UK	37	Male	25	I	NA
Ceccarelli et al., 2024	Case series	Italy	76	Male	29.2	II	NA
Ceccarelli et al., 2024	Case series	Italy	71	Male	26	II	NA
Ceccarelli et al., 2024	Case series	Italy	72	Female	24.3	III	NA
Ceccarelli et al., 2024	Case series	Italy	63	Male	27	III	NA
Ceccarelli et al., 2024	Case series	Italy	67	Female	28	II	NA
Ceccarelli et al., 2024	Case series	Italy	73	Male	24	II	NA
Ceccarelli et al., 2024	Case series	Italy	58	Male	23.7	II	NA
Ceccarelli et al., 2024	Case series	Italy	64	Female	27	II	NA
Ceccarelli et al., 2024	Case series	Italy	68	Female	24	II	NA
Ceccarelli et al., 2024	Case series	Italy	55	Male	24	II	NA
Ceccarelli et al., 2024	Case series	Italy	69	Female	25	II	NA
Ceccarelli et al., 2024	Case series	Italy	63	Male	24	II	NA
Ceccarelli et al., 2024	Case series	Italy	57	Male	27.4	II	NA
Ceccarelli et al., 2024	Case series	Italy	46	Female	24	II	NA
Ceccarelli et al., 2024	Case series	Italy	48	Female	25	II	NA
Petropoulou et al., 2025	Case series	Greece	91	Female	28	NA	Yes
Petropoulou et al., 2025	Case series	Greece	38	Male	26	NA	No
Petropoulou et al., 2025	Case series	Greece	55	Male	29.3	NA	Yes
Petropoulou et al., 2025	Case series	Greece	45	Male	34	NA	No
Petropoulou et al., 2025	Case series	Greece	81	Male	28	NA	No
Petropoulou et al., 2025	Case series	Greece	88	Male	27	NA	Yes
Petropoulou et al., 2025	Case series	Greece	74	Male	30	NA	Yes
Petropoulou et al., 2025	Case series	Greece	81	Male	25	NA	Yes
Petropoulou et al., 2025	Case series	Greece	72	Male	26	NA	Yes

NA = not applicable

### Surgical techniques performed in emergency setting

The most common emergency robotic procedures were right hemicolectomy for cancer (n = 9, 20%) and sigmoid colectomy for diverticular disease (n = 12, 26%). Each clinical case is reported in Supplementary File 2.

### Surgical delivery

Most patients (n = 44, 96%) underwent surgery in emergent or urgent setting at tertiary centres. All cases are reported in Supplementary File 2. When specified, all cases were performed using a da Vinci Surgical System (Intuitive Surgical, Inc, Sunnyvale, CA, USA): da Vinci Xi (n = 12, 33%), da Vinci X (n = 10, 27%), and da Vinci Si (n = 7, 19%). Nine studies did not mention the platform used. Among the 46 procedures, only one case [18] required conversion to open due to technical difficulty for an ileo-ileal intussusception recognized intraoperatively.

### Post-operative outcomes

Postoperative outcomes are summarized in Supplementary File 2. No Clavien-Dindo grade ≥ 3 complications were reported among all surgical procedures analysed. Thirty cases did not require either reoperation or readmission, while no data was available for the remaining cases. The study identified two major surgical groups: right hemicolectomies and sigmoid colectomies. Subgroup analyses were conducted for these two groups. Further analyses were not feasible due to the limited number and heterogeneity of the remaining procedures.

### Robotic right hemicolectomy

**Fig. 2:**
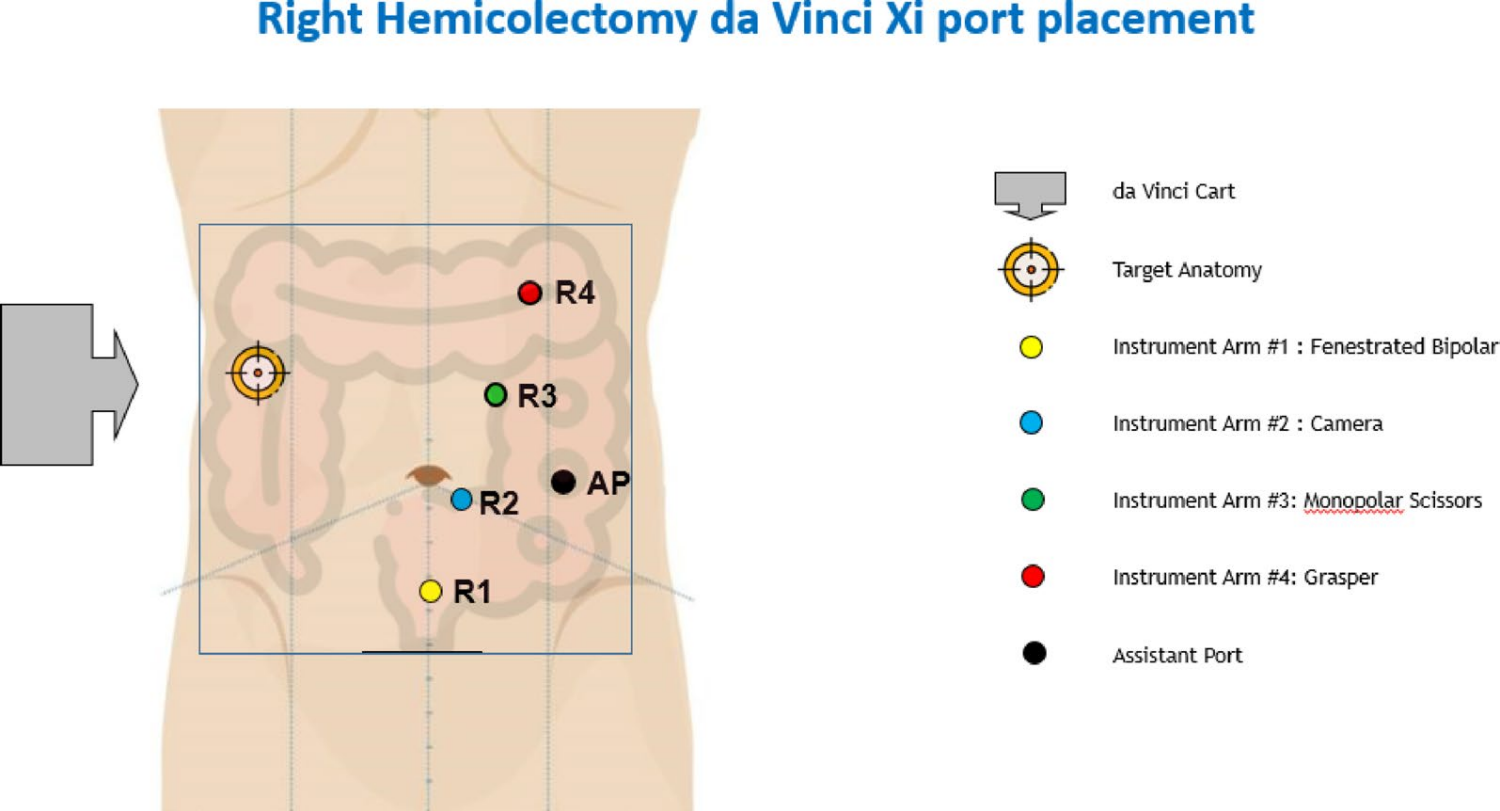
Robotic right hemicolectomy port placement

In most cases, robotic port placement followed a oblique linear setup (Fig. 2). For robotic right hemicolectomy in benign diseases, only one study reported operating time, which was 134 min [14]. In contrast, for malignant lesions, mean operating time was 241 ± 74 min. Estimated blood loss (EBL) was described in only one case during robotic right hemicolectomy for haemorrhagic colon cancer (50 mL) [22]. Regarding anastomotic techniques, intracorporeal semi-mechanical anastomoses were performed in two and seven cases for benign and malignant diseases, respectively. In one case, a double barrelled ileo-colostomy was created due to poor nutritional status [22], while in a separate case for benign disease, an extracorporeal anastomosis was performed [21]. Mean LOS for robotic right hemicolectomy for cancer was 5 ± 3 days, while LOS for benign cases was 5 days. Maertens et al. [11] reported resection margin status post CME in four cancer cases (all R0), with a mean lymph node harvest of 54 ± 13. Two CMEs were performed out of hours (one on a Monday, one on a Friday). In all cases, the team involved a expert robotic colorectal surgeon and an experienced theatre team and anaesthetist in robotic surgery.

Data on benign and malignant lesions are summarized in Tables 3 and 4, respectively. Follow-up data were limited with only Monsellato et al. [23] reporting a 9 days follow-up after right hemicolectomy for cancer and Jambhekar et al. describing a 21 months follow-up after right hemicolectomy for a Bochdalek hernia with ischaemic colon.

**Table 3:** Robotic right hemicolectomy for benign lesions

Outcomes	Observations (n)	Mean ± SD	Studies included (n)
Patient age, years	3	77 ± 3.6	3
BMI, kg/m^2^	2	28.5 ± 13.4	2
Operating time, min	1	NA	1
EBL, ml	0	NA	3
RBC transfusion rate	0	NA	3
Intraoperative complication rate	0	0	3
Conversion to open surgery rate	0	0	3
Intracorporeal anastomosis	2	–	2
Extracorporeal anastomosis	1	–	1
Stoma	0	0	3
Clavien-Dindo ≥ 3 complication rate	0	0	3
LOS, days	1	5	1
Reoperation rate	0	0	3
Readmission rate	0	0	3
Follow-up, months	0	21	3

BMI = Body Mass Index; EBL = Estimated Blood Loss; RBC = Red Blood Cell; LOS = Length of Stay

**Table 4:** Robotic right hemicolectomy for malignant lesions

Outcomes	Observations (n)	Mean ± SD	Studies included (n)
Patient age, years	11	71.9 ± 14.7	5
BMI, kg/m^2^	10	26.4 ± 3	4
Operating time, min	6	241.6 ± 7	3
EBL, ml	1	50	1
RBC transfusion rate	0	NA	5
Intraoperative complication rate	0	0	5
Conversion to open surgery rate	0	0	5
Intracorporeal anastomosis	6	–	4
Extracorporeal anastomosis	0	0	4
Stoma	1	0	4
Clavien-Dindo ≥ 3 complication rate	0	0	5
LOS, days	7	5 ± 3	4
Reoperation rate	0	0	5
Readmission rate	0	0	5
R0 resection margin rate	4	–	1
Follow-up, months	1	NA	1

BMI = Body Mass Index; EBL = Estimated Blood Loss; RBC = Red Blood Cell; LOS = Length of Stay

### Robotic sigmoid colectomy

**Fig. 3:**
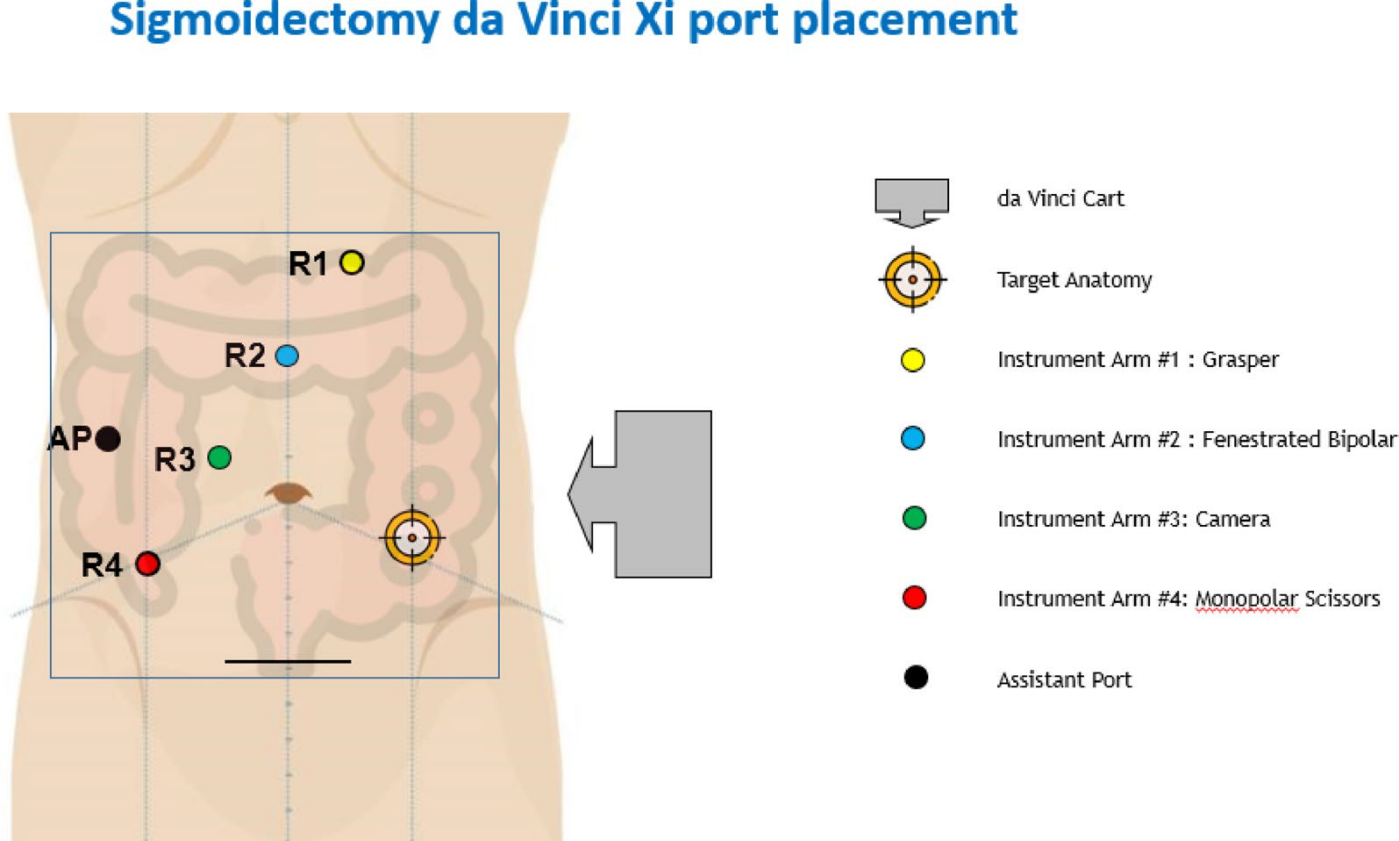
Robotic sigmoidectomy port placement

Most sigmoid colectomies were performed for diverticular disease. Only two studies [12, 14] reported the robotic port placement, which followed an oblique linear setup (Fig. 3). Robotic sigmoid colectomy had a mean operating time of 171.5 ± 3 min. An intracorporeal end-to-end hand-sewn anastomosis was reported in one case. Mean LOS was 4.6 ± 1 days. Follow-up data were reported only by Ceccarelli et al., with a mean duration of 21 ± 6 months. Data are summarized in Table 5. No intraoperative complications were recorded in any of the analysed reports.

**Table 5:** Robotic sigmoidectomy for diverticular disease

Outcomes	Observations (n)	Mean ± SD	Studies Included (n)
Patient age, years	10	67.3 ± 6	3
BMI, kg/m^2^	10	25 ± 3	3
Operating time, min	10	171.5 ± 3	3
EBL, ml	0	NA	3
RBC transfusion rate	0	NA	3
Intraoperative complication rate	0	0	3
Conversion to open surgery rate	0	0	3
Intracorporeal anastomosis	1	–	2
Extracorporeal anastomosis	0	0	2
Stoma	0	NA	2
Clavien-Dindo ≥ 3 complication rate	0	0	3
LOS, days	9	4.6 ± 1	2
Reoperation rate	0	0	3
Readmission rate	0	0	3
Follow-up, months	8	21 ± 6	1

BMI = Body Mass Index; EBL = Estimated Blood Loss; RBC = Red Blood Cell; LOS = Length of Stay

## Discussion

This study demonstrates that in emergency settings, robotic surgery may offer favourable outcomes in terms of operating time, intraoperative and postoperative complication rates, conversion to open surgery, LOS, as well as reoperation and readmission rates. Unfortunately, none of the included studies reported detailed perioperative and intraoperative set up. To the best of our knowledge, this is the first systematic review focusing only on the adoption of robotic approach for emergency colorectal surgery.

Over the past few decades, robotic surgery has been increasingly adopted globally for elective cases; however, its use in urgent and emergency settings remains limited, despite its potential advantages [24]. A study conducted in the United States from 2013 to 2021 demonstrated an increase in the use of robotics for colectomy in emergency settings, rising from 1.4 to 8.8%, with an average annual increase of 0.9% [25]. The benefits of robotic surgery in emergency settings have been highlighted, particularly in terms of lower conversion rates to open surgery (11.2% vs. 25.5%; OR 0.37 [95% CI 0.32–0.42]) and shorter LOS (− 0.48 days [95% CI − 0.60 to − 0.35]), when compared to laparoscopy [25].

Increased experience, proficiency, and expertise with the robotic approach among surgical teams, combined with greater access to robotic systems, have facilitated its adoption beyond elective procedures. Nevertheless, there has been hesitation in adopting robotics in urgent and emergency settings, reflecting a pattern similar to the initial historical experience with laparoscopy. To support the broader adoption of robotic surgery, hospitals should invest in comprehensive, practical training programs for surgeons, nurses, and all theatre staff, integrate robotics into standard surgical curricula, and ensure the availability of adequate numbers of trained surgical staff for robotic emergency surgery even during night shifts to prevent potential risk of burnout. A dedicated robotic platform for emergency surgery could also enhance its uptake during office hours. Moreover, Hospital Sterilisation and Decontamination Unit involvement should be obtained to optimize instrument availability and operational delivery in the event of consecutive cases being performed so that there is no disruption on the elective lists. With these measures in place, it is anticipated that operating times and costs could be optimized, with increasing volume, resulting in the routine use of robotic surgery in emergency settings.

Recently, the WSES position paper [26] highlighted the critical role of structured training programs in achieving proficiency in minimally invasive surgery (MIS) within the emergency setting. Five key opinion statements were outlined as foundational elements for a robust MIS training program: (1) the importance of understanding and progressing through the surgical learning curve; (2) the value of surgical proctoring and dedicated fellowships; (3) the relevance of training with surgical simulation systems and virtual reality platforms; (4) the necessity of maintaining a minimum caseload in emergency settings to ensure competence; and (5) the importance of training the entire surgical team, not just individual surgeons.

Anderson et al. [27] demonstrated the advantages of the robotic approach compared to laparoscopy, specifically for urgent subtotal colectomies in patients with ulcerative colitis. The robotic group showed better postoperative outcomes with a lower rate of complications (20% vs. 46%). Additionally, Grade III or higher complications occurred in 23% (3/13) of laparoscopic cases compared to 0% (0/5) in the robotic group. Reoperation rate was nil in the robotic group versus two cases in the laparoscopic group. Patients undergoing robotic surgery experienced earlier stoma function (1.4 ± 0.8 days vs. 2 ± 1.3 days, p = 0.1) and shorter LOS (3.4 ± 2 days vs. 4.6 ± 3.2 days, p = 0.3). There was no significant differences in intraoperative blood loss between the groups (80 ml vs. 75 ml, p = 0.9). However, the robotic approach was associated with a longer operative time (323.0 vs. 294.0 min, p = 0.3).

Charland et al. [28] analysed national trends and outcomes of robotic emergency general surgery in the United States, finding that the greatest increase in the use of the robotic surgical approach was observed in large bowel resection (from 0.09 to 20.1% between 2009 and 2021, *p* < 0.001). In addition, they reported significantly reduced rates of in-hospital mortality (0.66% vs. 1.28%, p = 0.03), complications (15.5% vs. 19.8%, *p* < 0.001), and median postoperative LOS (4 [3–6] days vs. 5 [3–7] days, *p* < 0.001) for large bowel resections performed robotically compared to laparoscopically. This highlights a growing adoption of the robotic approach in the USA, with significant benefits in perioperative outcomes.

Curfman et al. [29] conducted a retrospective multicentre review describing the advantages of the robotic approach in the emergency diverticulitis setting. They compared open (OS), laparoscopic (LS), and robotic surgery (RS). The study demonstrated the superiority of robotic surgery in terms of intensive care unit admission rates (OS 19.0%, RS 9.5%, p = 0.01), anastomotic leak rates (OS 4.4%, RS 0.8%, p = 0.04), and LOS (OS 9.9 days, RS 8.9 days, p = 0.05). Compared to LS, RS showed a reduction in anastomotic leak rates (LS 4.5% and RS 0.8%, p = 0.04) and a lower conversion rate to OS (LS 28.7% and RS 7.9%, p = 0.000005). However, RS was associated with increased operating time (LS 207 min, RS 262 min, OS 182 min).

Arnott et al. [30] analysed 6,880 non-elective colectomies for diverticulitis and found no significant differences between laparoscopic and robotic surgery regarding mortality, anastomotic leak, surgical site infection, reoperation, readmission, or LOS. Interestingly, the laparoscopic group exhibited a higher rate of postoperative sepsis (p = 0.001), which was attributed to factors such as higher BMI, Hispanic ethnicity, dependent functional status, higher ASA classification, and preoperative sepsis. The robotic group, however, showed increased bleeding rates (9.1% vs. 7.6%, p = 0.011) and a statistically longer mean operative time (237 vs. 175 min, *p* < 0.001), but a lower conversion rate to open surgery (11.5% vs. 28.3%, *p* < 0.001).

In a recent multicentre retrospective study, Grimsley et al. [31] reported higher costs associated with robotic procedures compared to laparoscopy, with no significant improvement in outcomes identified. This study included procedures such as cholecystectomies, appendectomies, and inguinal and ventral hernia repairs. The authors suggested that further research is needed to identify specific patient populations who might benefit from robotic surgery in emergency settings considering hospital finances and patients benefits.

Reinish et al. [32] also reported higher costs associated with robotic emergency procedures in their systematic review, although no specific data were available regarding colorectal surgery. Friedman [33] discusses the feasibility of robotic surgery in acute colorectal conditions, such as perforated diverticulitis and anastomotic leak. The author further emphasizes the importance of “after hours” access to the robotic platform and the presence of an appropriately trained surgical team.

The WSES reported that emergency setting should not be considered as an absolute contraindication for robotic surgery if a trained team is available [2]. Six key factors were identified for the successful implementation of robotic surgery in emergency settings: (1) the importance of surgeon expertise in an appropriately equipped operating room with trained nursing staff; (2) limiting the procedure to clinically stable patients; (3) leveraging the advantages of robotic surgery—such as field magnification, 3D stereoscopic vision, tremor filtration, and motion scaling—making it a viable option for difficult cases that might otherwise require open conversion from laparoscopy; (4) the potential future role of telementoring and telesurgery; (5) availability of the robotic platform during out-of-hours emergencies; and (6) the development of new modular robotic platforms, which could expand the applications of robotic surgery in emergency settings.

This study has few limitations. First, only cases involving major colorectal surgery were evaluated, excluding other colorectal procedures. Second, the analysed studies consisted primarily of case reports, case series, and video vignettes, many of which contained missing or low-quality data. It was not possible to assess the influence of patients’ surgical history on the planning of surgical strategies, as only two reports addressed this topic. Additionally, follow-up data was insufficient to evaluate postoperative outcomes, with only two authors providing it. Moreover, no study provided data on costs. Unfortunately, no study specified whether robotic surgeries were performed during regular hours or out-of-hours, which is an important aspect for future evaluation. Lastly, most cases were performed at tertiary centres with extensive robotic experience, limiting generalizability. Therefore, current evidence is insufficient to definitively prove the feasibility of robotic surgery in emergency settings, and prospective trials are warranted. Additionally, most of the research data on robotic applications in emergency settings comes from studies carried out in the USA. This represents a geographical limitation that reduces applicability to other healthcare systems.

To our knowledge, there is only one clinical trial on robotic emergency surgery (RObotic surgery in EMergency setting, ROEM) [34]. This is an observational, prospective, multicentre, international study analysing clinically stable adult patients undergoing robotic surgery for emergency treatment of acute pathologies, including diverticulitis in colorectal diseases. The primary aims of this trial is to evaluate the safety, feasibility, and cost-effectiveness of robotic surgery in emergency settings. Preliminary findings suggest that robotic surgery is feasible in stable patients in this context. Future directions should focus on optimizing costs, enhancing team training, and managing scheduling conflicts with elective surgeries.

## Conclusions

Robotic surgery in the emergency setting remains an upstream challenge and is currently offered to patients in a selective manner. This study depicts favourable outcomes following utilisation of robotic approach in emergency cases, including reduction in conversions to open surgery and intraoperative complications, with acceptable operating times and LOS, and no major postoperative complications, reoperations, or readmissions. However, further international, multicentre studies are needed to objectively confirm the role, safety, feasibility, economy and potential of emergency robotic surgery.

## Supplementary Information

Below is the link to the electronic supplementary material.

Supplementary Material 1

## Data Availability

The datasets used or analysed during the current study are available from the corresponding author upon reasonable request.

## Abbreviations

CME: Complete mesocolic excision
LOS: Length of stay
WSES: World society of emergency surgery
JBI: Joanna Briggs institute
BMI: Body mass index
ASA: American Society of Anesthesiologists
EBL: Estimated blood loss
OR: Odds ratio
CI: Confidential interval
MIS: Minimally invasive surgery
OS: Open surgery
LS: Laparoscopic surgery
RS: Robotic surgery
ROEM: RObotic surgery in EMergency setting

## References

[1] Gary S, GUTHART. Intuitive annual report. 2024.

[2] de Angelis N, Khan J, Marchegiani F, Bianchi G, Aisoni F, Alberti D, et al. Robotic surgery in emergency setting: 2021 WSES position paper. World J Emerg Surg WJES. 2022;17(1):4. 35057836 10.1186/s13017-022-00410-6 PMC8781145

[3] Pedraza R, Ragupathi M, Martinez T, Haas EM. Robotic-assisted laparoscopic primary repair of acute iatrogenic colonic perforation: case report. Int J Med Robot Comput Assist Surg MRCAS. 2012;8(3):375–8. 10.1002/rcs.1447 22736571

[4] Kalipershad SNR, Peristerakis I. The introduction of an emergency safety protocol coupled with simulation training in robotic surgery, has enabled a more cohesive and efficient response to emergencies. The Surgeon. 2022;20(3):151–6. 33947630 10.1016/j.surge.2021.03.007

[5] Shah SB, Chawla R, Rawal SK. Rapid undocking protocol for the da Vinci surgical robot during emergency situations. Indian J Anaesth. 2023;67(4):398–400. 37303879 10.4103/ija.ija_946_22 PMC10248886

[6] Anyomih TTK, Mehta A, Sackey D, Woo CA, Gyabaah EY, Jabulo M, et al. Robotic versus laparoscopic general surgery in the emergency setting: a systematic review. J Robot Surg. 5 July 2024;18(1):281. 10.1007/s11701-024-02016-3 38967691

[7] Liberati A, Altman DG, Tetzlaff J, Mulrow C, Gøtzsche PC, Ioannidis JPA, et al. The PRISMA statement for reporting systematic reviews and meta-analyses of studies that evaluate health care interventions: explanation and elaboration. PLoS Med. 21 July 2009;6(7):e1000100. 10.1371/journal.pmed.1000100 PMC2707010 19621070

[8] Ceccarelli G, Catena F, Avella P, Tian BW, Rondelli F, Guerra G, et al. Emergency robotic surgery: the experience of a single center and review of the literature. World J Emerg Surg WJES. 17 August 2024;19(1):28. 10.1186/s13017-024-00555-6 PMC11330055 39154016

[9] Dindo D, Demartines N, Clavien PA. Classification of surgical complications: a new proposal with evaluation in a cohort of 6336 patients and results of a survey. Ann Surg. 2004;240(2):205–13. 15273542 10.1097/01.sla.0000133083.54934.ae PMC1360123

[10] Moola S, Munn Z, Sears K, Sfetcu R, Currie M, Lisy K, et al. Conducting systematic reviews of association (etiology): The Joanna Briggs Institute’s approach. Int J Evid Based Healthc. 2015;13(3):163–9. 26262566 10.1097/XEB.0000000000000064

[11] Maertens V, Stefan S, Rawlinson E, Ball C, Gibbs P, Mercer S, et al. Emergency robotic colorectal surgery during COVID-19 pandemic: a retrospective case series study. Laparosc Endosc Robot Surg. 2022;5(2):57–60. 35342848 10.1016/j.lers.2022.03.001 PMC8938261

[12] Petropoulou T, Evangelou K, Polydorou A. Robotic-assisted surgery in emergency general surgery: a prospective, single-center, case series. Cureus. 2025;17(10):e93650. 10.7759/cureus.93650. 41181791 10.7759/cureus.93650 PMC12577499

[13] Kudsi OY, Gokcal F. Urgent robotic mesocolic excision for obstructing proximal transverse colon cancer—a video vignette. Colorectal Dis. 2019;21(9):1093–4. 31116470 10.1111/codi.14714

[14] Kudsi OY, Bou-Ayash N. Bleeding sigmoid diverticulosis—urgent stapleless totally robotic sigmoidectomy–a video vignette. Colorectal Dis. 2020;22(9):1205–1205. 32220039 10.1111/codi.15051

[15] Kudsi OY, Bou-Ayash N. Caecal volvulus—urgent totally robotic right colectomy—a video vignette. Colorectal Dis. 2020;22(10):1448–9. 32291898 10.1111/codi.15068

[16] Sneddon F, Richards C, Oliphant R. Emergent robotic assisted left hemicolectomy with intracorporeal anastomosis for descending colon cancer with retained colon capsule—a video vignette. Colorectal Dis. 2024;26(7):1476–1476. 38671578 10.1111/codi.17003

[17] de’Angelis N, Schena CA, Aisoni F, Marchegiani F. Robotic emergency Hartmann’s procedure for diverticulitis—A video vignette. Colorectal Dis. 2024;26(9):1768–9. 39073283 10.1111/codi.17107

[18] Alhammadi F, Prakash A, Alhashimi FM, Jaffar M, Ikram F, AlBastaki S. From an incidental lipoma to ileo-ileal intussusception in an adult: a case report. Int J Surg Case Rep. 2024;123:110164. 39178583 10.1016/j.ijscr.2024.110164 PMC11388267

[19] Alhomaid A, Sarwar MZ, Jawed R, Helal E, Buhl K. From chronic gallstone to acute ileus: a case report. Cureus. 2024;16(10):e72621. 10.7759/cureus.72621. 39610628 10.7759/cureus.72621 PMC11604247

[20] Smith D, Amiri F, Denning D. Gallstone ileus: a case report in a 74-year-old male. Am Surg. 2023;89(8):3612–3. 36951139 10.1177/00031348231167398

[21] Jambhekar A, Robinson S, Housman B, Nguyen J, Gu K, Nakhamiyayev V. Robotic repair of a right-sided Bochdalek hernia: a case report and literature review. J Robot Surg. 2018;12(2):351–5. 28500579 10.1007/s11701-017-0705-1

[22] Felli E, Brunetti F, Disabato M, Salloum C, Azoulay D, De’angelis N. Robotic right colectomy for hemorrhagic right colon cancer: A case report and review of the literature of minimally invasive urgent colectomy. World J Emerg Surg. 2014;9:32. 24791165 10.1186/1749-7922-9-32 PMC4005854

[23] Monsellato I, Lodin M, Priora F. Robotic right colectomy in a patient with ventriculoperitoneal shunt. Report of a case. Int J Surg Case Rep. 2019;59:58–62. 31103955 10.1016/j.ijscr.2019.05.018 PMC6601272

[24] Wong SW, Ang ZH, Yang PF, Crowe P. Robotic colorectal surgery and ergonomics. J Robot Surg aprile. 2022;16(2):241–6. 10.1007/s11701-021-01240-5. PubMed PMID: 33886064. 10.1007/s11701-021-01240-5 33886064

[25] Lunardi N, Abou-Zamzam A, Florecki KL, Chidambaram S, Shih IF, Kent AJ, et al. Robotic Technology in Emergency General Surgery Cases in the Era of Minimally Invasive Surgery. JAMA Surg. 2024;159(5):493. 10.1001/jamasurg.2024.0016. 38446451 10.1001/jamasurg.2024.0016 PMC10918578

[26] de’Angelis N, Marchegiani F, Schena CA, Khan J, Agnoletti V, Ansaloni L, et al. Training curriculum in minimally invasive emergency digestive surgery: 2022 WSES position paper. World J Emerg Surg. 2023;18(1):11. 10.1186/s13017-023-00476-w. 36707879 10.1186/s13017-023-00476-w PMC9883976

[27] Anderson M, Lynn P, Aydinli HH, Schwartzberg D, Bernstein M, Grucela A. Early experience with urgent robotic subtotal colectomy for severe acute ulcerative colitis has comparable perioperative outcomes to laparoscopic surgery. J Robot Surg. 2020;14(2):249–53. 10.1007/s11701-019-00968-5. PubMed PMID: 31076952. 31076952 10.1007/s11701-019-00968-5

[28] Charland N, Hadaya J, Mallick S, Tran Z, Cho NY, Le N, et al. National trends and outcomes of robotic emergency general surgery in the United States. Surgery. 2024;176(3):835–40. 10.1016/j.surg.2024.05.002. 38918109 10.1016/j.surg.2024.05.002

[29] Curfman KR, Jones IF, Conner JR, Neighorn CC, Wilson RK, Rashidi L. Robotic colorectal surgery in the emergent diverticulitis setting: is it safe? A review of large national database. Int J Colorectal Dis. 2023;38(1):142. 10.1007/s00384-023-04436-3. PubMed PMID: 37225935. 37225935 10.1007/s00384-023-04436-3

[30] Arnott SM, Arnautovic A, Haviland S, Ng M, Obias V. Safety of robotic surgical management of non-elective colectomies for diverticulitis compared to laparoscopic surgery. J Robot Surg. 2022;17(2):587–95. 10.1007/s11701-022-01452-3. 36048320 10.1007/s11701-022-01452-3

[31] Grimsley EA, Janjua HM, Herron T, Read MD, Lorch S, Cha JY, et al. Patient outcomes and cost in robotic emergency general surgery. J Robot Surg. 2023;17(6):2937–44. 10.1007/s11701-023-01739-z. 37856059 10.1007/s11701-023-01739-z

[32] Reinisch A, Liese J, Padberg W, Ulrich F. Robotic operations in urgent general surgery: a systematic review. J Robot Surg. 2022;17(2):275–90. 10.1007/s11701-022-01425-6. 35727485 10.1007/s11701-022-01425-6 PMC10076409

[33] Friedman G. Robotics for Acute Care in Colorectal Surgery. Clin Colon Rectal Surg. 2021;34(05):328–33. 10.1055/s-0041-1726448. 10.1055/s-0041-1726448 PMC8416323 34504404

[34] Milone M, Anoldo P, de’Angelis N, Coccolini F, Khan J, Kluger Y, et al. The role of RObotic surgery in EMergency setting (ROEM): protocol for a multicentre, observational, prospective international study on the use of robotic platform in emergency surgery. World J Emerg Surg. 2024;19(1):20. 10.1186/s13017-024-00542-x. 38835071 10.1186/s13017-024-00542-x PMC11149189

