# Supplementary material for: Robotic surgery in colorectal emergencies: a systematic review of current evidence
Source: World J Emerg Surg. 2026 May 8;21:40. doi: 10.1186/s13017-026-00685-z (PMC13321731; doi:10.1186/s13017-026-00685-z)
Supplement: Supplementary file 1 — Supplementary Material 1 [file 13017_2026_685_MOESM1_ESM.docx]

Supplementary File 1: PRISMA checklist

| **Section/topic** | | **#** | | **Checklist item** | | **Reported on page #** | |
| --- | --- | --- | --- | --- | --- | --- | --- |
| **TITLE** | | | | | |  | |
| Title | | 1 | | Identify the report as a systematic review, meta-analysis, or both. | | 1 | |
| **ABSTRACT** | | | | | |  | |
| Structured summary | | 2 | | Provide a structured summary including, as applicable: background; objectives; data sources; study eligibility criteria, participants, and interventions; study appraisal and synthesis methods; results; limitations; conclusions and implications of key findings; systematic review registration number. | | 3 | |
| **INTRODUCTION** | | | | | |  | |
| Rationale | | 3 | | Describe the rationale for the review in the context of what is already known. | | 4 | |
| Objectives | | 4 | | Provide an explicit statement of questions being addressed with reference to participants, interventions, comparisons, outcomes, and study design (PICOS). | | 4 and 5 | |
| **METHODS** | | | | | |  | |
| Protocol and registration | | 5 | | Indicate if a review protocol exists, if and where it can be accessed (e.g., Web address), and, if available, provide registration information including registration number. | | 5 | |
| Eligibility criteria | | 6 | | Specify study characteristics (e.g., PICOS, length of follow-up) and report characteristics (e.g., years considered, language, publication status) used as criteria for eligibility, giving rationale. | | 5 and 6 | |
| Information sources | | 7 | | Describe all information sources (e.g., databases with dates of coverage, contact with study authors to identify additional studies) in the search and date last searched. | | 5, 6 and 7 | |
| Search | | 8 | | Present full electronic search strategy for at least one database, including any limits used, such that it could be repeated. | | 5 | |
| Study selection | | 9 | | State the process for selecting studies (i.e., screening, eligibility, included in systematic review, and, if applicable, included in the meta-analysis). | | 7 and Figure n.1 | |
| Data collection process | | 10 | | Describe method of data extraction from reports (e.g., piloted forms, independently, in duplicate) and any processes for obtaining and confirming data from investigators. | | 7 | |
| Data items | | 11 | | List and define all variables for which data were sought (e.g., PICOS, funding sources) and any assumptions and simplifications made. | | 5 and 6 | |
| Risk of bias in individual studies | | 12 | | Describe methods used for assessing risk of bias of individual studies (including specification of whether this was done at the study or outcome level), and how this information is to be used in any data synthesis. | | 6 | |
| Summary measures | | 13 | | State the principal summary measures (e.g., risk ratio, difference in means). | | Table 3, 4 and 5 | |
| Synthesis of results | | 14 | | Describe the methods of handling data and combining results of studies, if done, including measures of consistency (e.g., I^2^) for each meta-analysis. | | Table 3, 4 and 5 | |
| Section/topic | | # | | Checklist item | | Reported on page # | |
| Risk of bias across studies | | 15 | | Specify any assessment of risk of bias that may affect the cumulative evidence (e.g., publication bias, selective reporting within studies). | | 6 and table 1 | |
| Additional analyses | | 16 | | Describe methods of additional analyses (e.g., sensitivity or subgroup analyses, meta-regression), if done, indicating which were pre-specified. | | Not available | |
| **RESULTS** | | | | | |  | |
| Study selection | | 17 | | Give numbers of studies screened, assessed for eligibility, and included in the review, with reasons for exclusions at each stage, ideally with a flow diagram. | | 7 | |
| Study characteristics | | 18 | | For each study, present characteristics for which data were extracted (e.g., study size, PICOS, follow-up period) and provide the citations. | | 7 and table 3, 4 and 5 | |
| Risk of bias within studies | | 19 | | Present data on risk of bias of each study and, if available, any outcome level assessment (see item 12). | | 6 and table 1 | |
| Results of individual studies | | 20 | | For all outcomes considered (benefits or harms), present, for each study: (a) simple summary data for each intervention group (b) effect estimates and confidence intervals, ideally with a forest plot. | | Table n.3 | |
| Synthesis of results | | 21 | | Present results of each meta-analysis done, including confidence intervals and measures of consistency. | | 7, 8 and 9 | |
| Risk of bias across studies | | 22 | | Present results of any assessment of risk of bias across studies (see Item 15). | | 6 and table 1 | |
| Additional analysis | | 23 | | Give results of additional analyses, if done (e.g., sensitivity or subgroup analyses, meta-regression [see Item 16]). | | Not available | |
| **DISCUSSION** | | | | | |  | |
| Summary of evidence | | 24 | | Summarize the main findings including the strength of evidence for each main outcome; consider their relevance to key groups (e.g., healthcare providers, users, and policy makers). | | 10, 11, 12 and 13 | |
| Limitations | | 25 | | Discuss limitations at study and outcome level (e.g., risk of bias), and at review-level (e.g., incomplete retrieval of identified research, reporting bias). | | 13 and 14 | |
| Conclusions | | 26 | | Provide a general interpretation of the results in the context of other evidence, and implications for future research. | | 14 | |
| **FUNDING** | | | | | |  | |
| Funding | | 27 | | Describe sources of funding for the systematic review and other support (e.g., supply of data); role of funders for the systematic review. | | 14 | |

Supplementary File 2: Postoperative outcomes.

| **Publication, year** | **Diagnosys** | **Surgical techniques** | **Surgery performed out of hours?** | **Type of Hospital** | **Robotic platform** | **Port number** | **Port placement** | **Operative time, mins** | **EBL** | **Intraoperative complications** | **conversion** | **Intracorporeal/extracorporeal anastomoys** | **Stoma** | **LOS days** | **Postoperative complications** | **Reoperation** | **Readmission** | **R0** | **Follow-up months** |
| --- | --- | --- | --- | --- | --- | --- | --- | --- | --- | --- | --- | --- | --- | --- | --- | --- | --- | --- | --- |
| Pedraza et al., 2012 | Iatrogenic sigmoid perforation | Sigmoid suture | NA | Tertiary | Da vinci | 4 | -camera arm periumbilical region -arms 1, 2 and 3 right lower quadrant, left upper quadrant and left lower quadrant | 135 | NA | NO | NO | NA (suture) | NO | 4 | NO | NO | NO |  | 1 |
| Smith et al., 2023 | Gallstone ileus with ostruction | Enterotomy | NA | Tertiary | NA | 3 | upper abdominal port placement ? | NA | NA | NO | NO | NA | NO | 3 | NO | NO | NO |  | NA |
| Alhammadi et al., 2024 | Lipoma with ileo-ileal intussusception | Ileal resection | NA | Tertiary | Da Vinci | NA | NA | NA | NA | NO | YES | extracoporeal side to side mecanical ileo-ileal | NO | 3 | NO | NO | NO |  | 2 W |
| Alhomaid et al., 2024 | Chronic Gallstone with Acute Ileus | Enterotomy | NA | Secondary | NA | NA | NA | NA | NA | NO | NO | NA | NO | NA | NO | NO | NO |  | NA |
| Felli et al., 2014 | Hemorrhagic right colon cancer | Right hemicolectomy | NA | Tertiary | Da vinci | 3+ Assistant port | Arciform left side | 150 | 50 | NO | NO | NA (ileocolostomy) | YES ileocolostomy | 6 | NO | NO | NO | NA | NA |
| Jambhekar et al., 2018 | Bochdalek hernia with ischemia of herniated colon | Right hemicolectomy and diaphragmatic hernia repair | NA | Secondary | Da vinci Si | 4 + Assistant port | Arciform ( camera port above the umbilicus (Cam), three 8-mm ports : left upper quadrant- midclavicular line at the time of the umbilicus - in the right upper quadrant below the costal margin; assistant port between ports R2 and R3 | NA | NA | NO | NO | extracoporeal side to side ileocolic | NO | 5 | NO | NO | NO |  | 21 |
| Monsellato et al., 2019 | Ulcerated tumor of the right colon | Right hemicolectomy | NA | Tertiary | NA | 4 + Assistant port | Arciform left side | 280 | NA | NO | NO | Intracorporeal | NO | 5 | NO | NO | NO | NA | 9D |
| Kudsi et al., 2019 | Obstructing proximal transverse colon cancer | Extended Right hemicolectomy with CME | NA | Tertiary | Da vinci | 4 | Da vinci R hemi port placement | NA | NA | NO | NO | intracorporeal semi mecanical | NO | 5 | NO | NO | NO | NA | NA |
| Kudsi et al., 2020 | Bleeding diverticulitis | Sigmoidectomy | NA | Tertiary | Da vinci Xi | 4 | Da vinci L hemi port placement | 145 | NA | NO | NO | intracorporeal end-to-end hand sewn | NO | NA | NO | NO | NO |  | NA |
| Kudsi et al., 2020 | Caecal volvulus | Right hemicolectomy | NA | Tertiary | Da vinci Xi | 4 | Da vinci R hemi port placement | 134 | NA | NO | NO | intracorporeal semi mecanical | NO | NA | NO | NO | NO |  | NA |
| De Angelis et al., 2024 | Diverticulitis + colovescical fistula complicated by urosepsis | Hartmann procedure | NA | Tertiary | Da vinci | 4 + Assistant port | Da vinci L hemi port placement | NA | NA | NO | NO | NA | YES colostomy | 4 | NO | NO | NO |  | NA |
| Sneddon et al., 2024 | Obstructing colon cancer with retained colon capsule | Left hemicolectomy | NA | Tertiary | Da vinci Xi | 4 + Assistant port | Da vinci L hemi port placement | NA | NA | NO | NO | intracorporeal end-to-end hand sewn | NO | 3 | NO | NA | NA | NA | NA |
| Maertens et al., 2022 | Diverticulitis + colovescical fistula | Anterior resection | NO | Tertiary | Da vinci X | 4 + Assistant port | Da vinci L hemi port placement | 249 ± 117 | NA | NO | NO | NA (Hartmann) | YES colostomy | 9.4 (5-22) | 2 CD I, 2 CDII , 6 none | NO | NO |  | NA |
| Maertens et al., 2022 | Complicated Chron's disease with obstruction | Right hemicolectomy and repair of bladder fistula | NO | Tertiary | Da vinci X | 4 + Assistant port | Da vinci R hemi port placement | 249 ± 117 | NA | NO | NO | intracorporeal semi mecanic | NO | 9.4 (5-22) | 3 CD I, 2 CDII , 6 none | NO | NO |  | NA |
| Maertens et al., 2022 | Ulcerative colitis with fulminant colitis | Subtotalcolectomy and definitive ileostomy | YES | Tertiary | Da vinci X | 4 + Assistant port | NA | 249 ± 117 | NA | NO | NO | NA (definitive ileostomy) | YES ileostomy | 9.4 (5-22) | 4 CD I, 2 CDII , 6 none | NO | NO |  | NA |
| Maertens et al., 2022 | Perforated diverticular disease (Hinchey 3) | Anterior resection | YES | Tertiary | Da vinci X | 4 + Assistant port | Da vinci L hemi port placement | 249 ± 117 | NA | NO | NO | intracorporeal semi mecanical | NO | 9.4 (5-22) | 5 CD I, 2 CDII , 6 none | NO | NO |  | NA |
| Maertens et al., 2022 | Perforated diverticular disease (Hinchey 3) | Anterior resection | YES | Tertiary | Da vinci X | 4 + Assistant port | Da vinci L hemi port placement | 249 ± 117 | NA | NO | NO | intracorporeal semi mecanical | NO | 9.4(5-22) | 2 CD I, 2 CDII , 6 none | NO | NO |  | NA |
| Maertens et al., 2022 | Obstructed parastomal hernia with strangulated small bowel | Bowel resection and parastomal hernia repair | NA | Tertiary | Da vinci X | 4 + Assistant port | NA | 249 ± 117 | NA | NO | NO | intracorporeal semi mecanical | NO | 9.4(5-22) | 3 CD I, 2 CDII , 6 none | NO | NO |  | NA |
| Maertens et al., 2022 | Perforated right colon cancer | Right hemicolectomy with CME | YES | Tertiary | Da vinci X | 4 + Assistant port | Da vinci R hemi port placement | 249 ± 117 | NA | NO | NO | intracorporeal semi mecanical | NO | 9.4(5-22) | 4 CD I, 2 CDII , 6 none | NO | NO | R0 +mean lymphnode harvest 54 ± 13 | NA |
| Maertens et al., 2022 | Intussusception right colon cancer | Right hemicolectomy with CME | NO | Tertiary | Da vinci X | 4 + Assistant port | Da vinci R hemi port placement | 249 ± 117 | NA | NO | NO | intracorporeal semi mecanical | NO | 9.4(5-22) | 5 CD I, 2 CDII , 6 none | NO | NO | R0 +mean lymphnode harvest 54 ± 13 | NA |
| Maertens et al., 2022 | Obstruction and perforated transverse colon cancer | Extended Right hemicolectomy with CME | YES | Tertiary | Da vinci X | 4 + Assistant port | Da vinci R hemi port placement | 249 ± 117 | NA | NO | NO | intracorporeal semi mecanical | NO | 9.4(5-22) | 6 CD I, 2 CDII , 6 none | NO | NO | R0 +mean lymphnode harvest 54 ± 13 | NA |
| Maertens et al., 2022 | Locally advanced obstructing right colon cancer | Right hemicolectomy with CME | YES | Tertiary | Da vinci X | 4 + Assistant port | Da vinci R hemi port placement | 249 ± 117 | NA | NO | NO | intracorporeal semi mecanical | NO | 9.4(5-22) | 7 CD I, 2 CDII , 6 none | NO | NO | R0 +mean lymphnode harvest 54 ± 13 | NA |
| Ceccarelli et al., 2024 | Colovescical fistula | Sigmoidectomy and repair of bladder fistula | NA | Tertiary | Da vinci Xi | NA | NA | 180 | NA | NO | NO | NA | NA | 5 | CD I | NA | NA |  | 26 |
| Ceccarelli et al., 2024 | Colovescical fistula | Sigmoidectomy and repair of bladder fistula | NA | Tertiary | Da vinci Xi | NA | NA | 170 | NA | NO | NO | NA | NA | 6 | NO | NA | NA |  | 24 |
| Ceccarelli et al., 2024 | Colovescical fistula | Hartmann and repair of bladder fistula | NA | Tertiary | Da vinci Xi | NA | NA | 310 | NA | NO | NO | NA | YES colostomy | 5 | CD II | NA | NA |  | 7 |
| Ceccarelli et al., 2024 | Acute diverticulitis | Left hemicolectomy | NA | Tertiary | Da vinci Si | NA | NA | 170 | NA | NO | NO | NA | NA | 5 | NO | NA | NA |  | 36 |
| Ceccarelli et al., 2024 | Acute diverticulitis | Sigmoidectomy | NA | Tertiary | Da vinci Si | NA | NA | 190 | NA | NO | NO | NA | NA | 5 | NO | NA | NA |  | 28 |
| Ceccarelli et al., 2024 | Acute diverticulitis | Sigmoidectomy | NA | Tertiary | Da vinci Si | NA | NA | 185 | NA | NO | NO | NA | NA | 4 | CD I | NA | NA |  | 24 |
| Ceccarelli et al., 2024 | Acute diverticulitis + abscess | Sigmoidectomy and omentoplasty | NA | Tertiary | Da vinci Xi | NA | NA | 210 | NA | NO | NO | NA | NA | 6 | CD I | NA | NA |  | 10 |
| Ceccarelli et al., 2024 | Acute diverticulitis | Sigmoidectomy | NA | Tertiary | Da vinci Xi | NA | NA | 160 | NA | NO | NO | NA | NA | 5 | NO | NA | NA |  | 24 |
| Ceccarelli et al., 2024 | Acute diverticulitis | Sigmoidectomy | NA | Tertiary | Da vinci Xi | NA | NA | 170 | NA | NO | NO | NA | NA | 4 | NO | NA | NA |  | 16 |
| Ceccarelli et al., 2024 | Acute diverticulitis | Sigmoidectomy | NA | Tertiary | Da vinci Xi | NA | NA | 125 | NA | NO | NO | NA | NA | 5 | NO | NA | NA |  | 16 |
| Ceccarelli et al., 2024 | Acute diverticulitis | Left hemicolectomy | NA | Tertiary | Da vinci Xi | NA | NA | 160 | NA | NO | NO | NA | NA | 5 | NO | NA | NA |  | 10 |
| Ceccarelli et al., 2024 | Ostruction for ileocecal valve stricture in Chron's disease | Ileocaecal resection | NA | Tertiary | Da vinci Xi | NA | NA | 135 | NA | NO | NO | NA | NA | 5 | NO | NA | NA |  | 42 |
| Ceccarelli et al., 2024 | Obstruction for ileocecal valve stricture in Chron's disease | Ileocaecal resection | NA | Tertiary | Da vinci Si | NA | NA | 180 | NA | NO | NO | NA | NA | 4 | NO | NA | NA |  | 28 |
| Ceccarelli et al., 2024 | Bowel endometriosis | Sigmoidectomy | NA | Tertiary | Da vinci Si | NA | NA | 165 | NA | NO | NO | NA | NA | 5 | NO | NA | NA | NA | 42 |
| Ceccarelli et al., 2024 | Bowel endometriosis | Ileocaecal resection | NA | Tertiary | Da vinci Si | NA | NA | 185 | NA | NO | NO | NA | NA | 6 | NO | NA | NA | NA | 38 |
| Petropoulou et al, 2025 | Obstructing transverse colon tumor | Transverse colectomy | NA | Tertiary | NA | 4+Assistant port | standardized four-arm configuration for colectomies | 180 | NA | NO | NO | NA | NA | 2 | NO | NO | NO | NA | NA |
| Petropoulou et al, 2025 | Obstructing upper rectal tumor | Anterior resection | NA | Tertiary | NA | 4+Assistant port | standardized four-arm configuration for colectomies | 180 | NA | ureteric injury | NO | NA | NA | 2 | NO | NO | NO | NA | NA |
| Petropoulou et al, 2025 | Obstructing sigmoid colon tumor | Right colectomy, sigmoidectomy, extensive adhesiolysis, and resection of recurrent liposarcoma tumours | NA | Tertiary | NA | 4+Assistant port | standardized four-arm configuration for colectomies | 360 | NA | NO | NO | NA | NA | 11 | Surgical site infection | NO | NO | NA | NA |
| Petropoulou et al, 2025 | Acute diverticulitis | Anterior resection | NA | Tertiary | NA | 4+Assistant port | standardized four-arm configuration for colectomies | 240 | NA | NO | NO | NA | NA | 2 | NO | NO | NO | NA | NA |
| Petropoulou et al, 2025 | Obstructing cecal tumor | Right hemicolectomy | NA | Tertiary | NA | 4+Assistant port | standardized four-arm configuration for colectomies | 240 | NA | NO | NO | NA | NA | 2 | NO | NO | NO | NA | NA |
| Petropoulou et al, 2025 | Obstructing ascending colon tumor | Right hemicolectomy | NA | Tertiary | NA | 4+Assistant port | standardized four-arm configuration for colectomies | 180 | NA | NO | NO | NA | NA | 4 | NO | NO | NO | NA | NA |
| Petropoulou et al, 2025 | Obstructing rectal tumor | Anterior resection and ileostomy | NA | Tertiary | NA | 4+Assistant port | standardized four-arm configuration for colectomies | 240 | NA | NO | NO | NA | NA | 6 | NO | NO | NO | NA | NA |
| Petropoulou et al, 2025 | Bleeding ascending colon tumor | Right hemicolectomy and ileostomy | NA | Tertiary | NA | 4+Assistant port | standardized four-arm configuration for colectomies | 240 | NA | NO | NO | NA | NA | 2 | NO | NO | NO | NA | NA |
| Petropoulou et al, 2025 | Perforated diverticulitis | Hartmann’s procedure and intraabdominal abscess drainage | NA | Tertiary | NA | 4+Assistant port | standardized four-arm configuration for colectomies | 180 | NA | NO | NO | NA | NA | 2 | NO | NO | NO | NA | NA |
